# The Effects of Magnesium Sulfate with Lidocaine for Infraclavicular Brachial Plexus Block for Upper Extremity Surgeries

**DOI:** 10.1055/s-0040-1715578

**Published:** 2020-11-06

**Authors:** Siavash Beiranvand, Arash Karimi, Majid Haghighat Shoar, Maryam Baghizadeh Baghdashti

**Affiliations:** 1 Department of Anesthesiology, Faculty of Medicine, Lorestan University of Medical Sciences, Khorramabad, Iran

**Keywords:** magnesium sulfate, anesthetic, infraclavicular, brachial plexus, pain, lidocaine

## Abstract

**Background** An addition of analgesic to anesthetic agents is likely to increase the effects of anesthesia and reduce associated adverse outcomes. Several adjuvants are studied in this regard. The aim of this study is to investigate the effects of adding a magnesium adjunct to lidocaine for the induction of infraclavicular block.

**Methods** Patients referred to Shohada Ashayer Hospital, Khorramabad, for wrist and hand surgery were enrolled in this study. The intervention/case group included patients who received 18 mL lidocaine (2%) + 2 mL magnesium sulfate (50%), 10 mL normal saline; control group: 18 mL lidocaine (2%) + 12 mL of normal saline. After the induction of ultrasound-guided infraclavicular block, parameters such as duration of reach with respect to complete sensory and motor block, hemodynamic parameters (hypotension and bradycardia), and postoperative pain, using visual analogue scale criteria, were measured. The obtained data were analyzed using a Bayesian path analysis model.

**Results** A total of 30 patients were included in each group. In the case group, sensory and motor block was achieved for 12.136 ± 4.96 and 13 ± 3.589 minutes more than those in the control group. The duration of sedation and immobilization was 2.57 ± 0.764 minute and 4.66 ± 0.909 minutes lengthier in the case group. Regarding the hemodynamic parameters, blood pressure was 0.217 ± 5.031 and 1.59 ± 5.14 units lower in the case group, immediately following the block and the surgery. Similarly, heart rate was 0.776 ± 4.548 and 0.39 ± 3.987 units higher in the case group, after 30 minutes and 2 hours of the procedure. A decrease in the pain was seen at 8, 10, and 12 hours after the surgery, as compared with the control group. An addition of magnesium to lidocaine for infraclavicular block resulted in a significantly longer sedation and immobilization period and decreased postoperative pain at 12 hours.

**Conclusion** Heart rate and blood pressure did not decrease significantly in the case group. It can be concluded that addition of magnesium sulfate to lidocaine can produce better anesthetic and analgesic outcomes with low-to-no adverse effects.

## Introduction

Orthopedic procedures such as those related to hand, wrist, and forearm procedures, despite being minor surgical procedures, are associated with a great amount of postoperative pain. Infraclavicular neural network block is useful in causing prolonged and effective postoperative analgesia in these patients. 1

Ultrasound-guided infraclavicular brachial plexus nerve block has been practiced recently, where different approaches such as transverse, posterior costoclavicular, medial, distal, and proximal approaches are commonly used. 2 The distal approach is conventionally used; however, the proximal method is likely to be associated with the lessened use of anesthetic agents. 3 Nonetheless, pneumothorax and neuraxial spread are some common complications associated with the ultrasound guide. 4 5

Lidocaine is a local anesthetic rapid-acting agent that is used for the blockade of motor and sensory fibers for up to 1.5 hours. 6 Several adjuncts have been investigated to elevate the analgesic response of lidocaine for infraclavicular block. 7

The effect of magnesium was first recognized for the treatment of arrhythmia and preeclampsia, and its effect on anesthesia and analgesia has recently been recognized. 8 9 Magnesium sulfate has also been used as an adjunct to anesthesia in recent years. It is also an effective analgesic agent for perioperative pain. 10 11 Researches have also reported that the intraoperative use of magnesium is characterized by a reduced use of anesthetics and muscle relaxants. 12 Furthermore, opioid use, postoperative nausea and vomiting, hypertension, and shivering have met a decreased trend with the use of magnesium sulfate. 13 14

This study is designed to evaluate the effects of addition of magnesium sulfate to lidocaine for infraclavicular neural network block in pain control during and after hand, wrist, and forearm surgery in patients referred to Shohada Ashayer Hospital in 2018.

## Methods

The aim of this study was to evaluate the effects of magnesium sulfate supplementation with lidocaine for infraclavicular nerve block for postoperative pain management following hand, wrist, and forearm surgery in patients referred to Shohada Nassir Hospital, Khorramabad between February 2018 and 2019. Patients undergoing the procedure were selected by a simple sampling method, where patients were randomly divided into two groups; group A included the patients receiving magnesium sulfate with 2% of lidocaine and group B included the patients administered saline with 2% lidocaine. Patients aged 18 to 85 years, ASA I–II class, having consent to participate in the study were included in the study. Patients with contraindication of brachial nerve block (allergy to local anesthesia, local infection at injection site, and coagulopathy), traumatic nerve injury of upper limb, history of opioid abuse, alcohol and drug abuse, recent chronic analgesic treatment, celiac and meningitis, allergic to lidocaine, peripheral neuropathy, neuromuscular disease, pregnancy and lactation, specific psychosis disorders, cognitive impairments, and those who disagreed to participate were excluded from the study. After receiving a written consent, detailed explanations of the study were provided to the patients. The patients were allotted a unique code which was only known to the nurse in-charge of the anesthesiology unit.

The infraclavicular neural network block was performed by the anesthesiologist. Noninvasive monitoring (blood pressure, heart rate) was performed and Ringer's solution was infused. All patients received premedication 0.5 mg/kg midazolam and 2.2 mg/kg fentanyl, prior to the block. Under the guidance of ultrasound (Ezono 3000, Germany), using a linear ultrasound probe, the infraclavicular neural network was identified and the in-plane method with SonoPlex needle (22G) was used to inject the following as per the group allocations: case group: 18 mL lidocaine (2%) + 2 mL magnesium sulfate (50%), 10 mL normal saline; control group: 18 mL lidocaine (2%) + 12 mL of normal saline.

The patient's block was placed in a supine position, where the patient's arm was abducted to reduce the depth between the plexus and the skin. The cords of the brachial plexus are seen as hyperechoic circles bordered by axillary artery. A needle was inserted using the in-plane method, from the inferior of the ultrasound transducer, 1 cm into the skin. After reaching the artery, anesthetic agents were induced.

Following the block, the patients were evaluated for hemodynamic changes and block complications, such as pneumothorax, hypotension, bradycardia, and hematoma. The decline in sensory and motor activity was assessed after every 2 minutes, following the block, until complete sensory block was achieved. Furthermore, during the surgery, sensory and motor assessment was performed after every 5 minutes, during the first 30 minutes of the surgical procedure. Subsequently, motor and sensory activity was monitored after every 15 minutes until the end of the surgery. In any case, where anesthesia failure was seen, 30 minutes after the bock, it was marked as the infraclavicular block failure. The magnitude of the motor block was measured with Bromage scores of 16:1 = complete leg movement, 2 = partial movement, 3 = relative movement, and 4 = complete immobility.

Sensory block was measured by a pinprick test: 0 = no sensation, 1 = sensory loss, and 2 = no sensory change. The duration of the sensory block is the period between the end of local anesthetic administration and normal sensory return. The duration of the locomotor block is the period between the end of local anesthetic administration and complete motor function reversal. The patient's blood pressure and heart rate were recorded before the block, 30 minutes after the end of the injection, at the end of surgery, and 2 hours after the surgery. If the blood pressure dropped below 20% of the baseline blood pressure, 5 mg of ephedrine was injected. When the heart rate decreased below 50/minute, 0.5 mg of atropine was injected. Postoperative pain was measured at 2, 4, 6, 8, 10, and 12 hours using the visual analogue scale criterion where 0 indicated no pain and 10 indicated worst pain imaginable. The pain was evaluated based on the type of the surgery performed and patients were educated accordingly regarding the perception of the pain. Side effects such as nausea, vomiting, bradycardia, hypotension, and itching at 4, 8, and 12 hours after surgery were also evaluated. The duration of the sensory block was the primary finding, whereas the duration of the motor block, onset of sensory and motor block, total opioid use, and postoperative pain score were secondary outcomes of the study. The obtained data were recorded in the evaluation form and were assessed using SPSS V. 21.

To investigate the effect of intervention (group A: lidocaine with magnesium sulfate; group B: saline with lidocaine) on the dependent variables in the neural network block subgroup (duration of anesthesia, onset anesthesia time, and onset immobility time), biological factors (blood pressure and heart rate), and pain at different times, a Bayesian path analysis model was used that could help us determine the significant correlation between the dependent variables in each subgroup. Since the correlation between the dependent variables is significant, concurrent statistical inferences were required, to determine the effects of the intervention on the dependent factors, for which route analysis was exploited. In Bayesian inference, the validity intervals, instead of *p* -value, are used to examine the significance of the effect of the intervention variable on the dependent variables. If the interval of validity is zero, the intervention was known to have no significant effect.

## Results

The results of the study regarding the effects of intervention on dependent variables in the neural network block subgroup using Bayesian path analysis are shown below.

### Determination and Comparison of Sensory Block Duration in the Two Study Groups

According to the obtained validity interval, it can be concluded that the intervention had a significant effect on the duration of anesthesia, as the duration of anesthesia for the target group patients was approximately 12.13 ± 4.96 minutes longer than the control group. In addition, variables such as age, gender, and body mass index (BMI) have significant effects on the duration of anesthesia ( Table 1 ).

**Table 1:** Bayesian path analysis output to investigate the effect of intervention, age, gender, and BMI on anesthesia duration

Variable	Estimate	SE	Lower CI	Upper CI
Group (ref = control)	12.136	4.96	2.409	21.979
Age	0.529	0.138	0.255	0.799
Sex (ref = male)	12.49	4.839	3.202	22.112
BMI	0.132	0.019	0.096	0.17

Abbreviations: BMI, body mass index; CI, confidence interval; SE, standard error.

### Determination and Comparison of Motor Block Duration in the Two Study Groups

According to the obtained interval of validity, it can be deduced that the intervention had a significant effect on the duration of immobility, as the duration of anesthesia for the target group patients was approximately 13.14 ± 3.589 minutes longer than the control group. In addition, variables such as age, gender, and BMI have significant effects on the duration of immobility ( Table 2 ).

**Table 2:** Bayesian path analysis output to investigate the effect of intervention, age, gender, and BMI on immobilization duration

Variable	Estimate	SE	Lower CI	Upper CI
Group (ref = control)	13.14	3.589	6.117	20.092
Age	0.446	0.095	0.26	0.635
Sex (ref = male)	9.258	3.484	2.02	15.699
BMI	0.105	0.013	0.08	0.132

Abbreviations: BMI, body mass index; CI, confidence interval; SE, standard error.

### Determination and Comparison of Time to Anesthesia in the Two Study Groups

The mean time to anesthetize for the case group was approximately 2.57 ± 0.764 minutes longer than the control group. Considering the validity interval, it can be concluded that the intervention had a significant effect on the time to anesthetize. In addition, age and BMI had a significant effect, as well. However, no such difference was reported in terms of gender.

### Determination and Comparison of the Onset Time of Immobility in the Two Study Groups

**Fig. 1:**
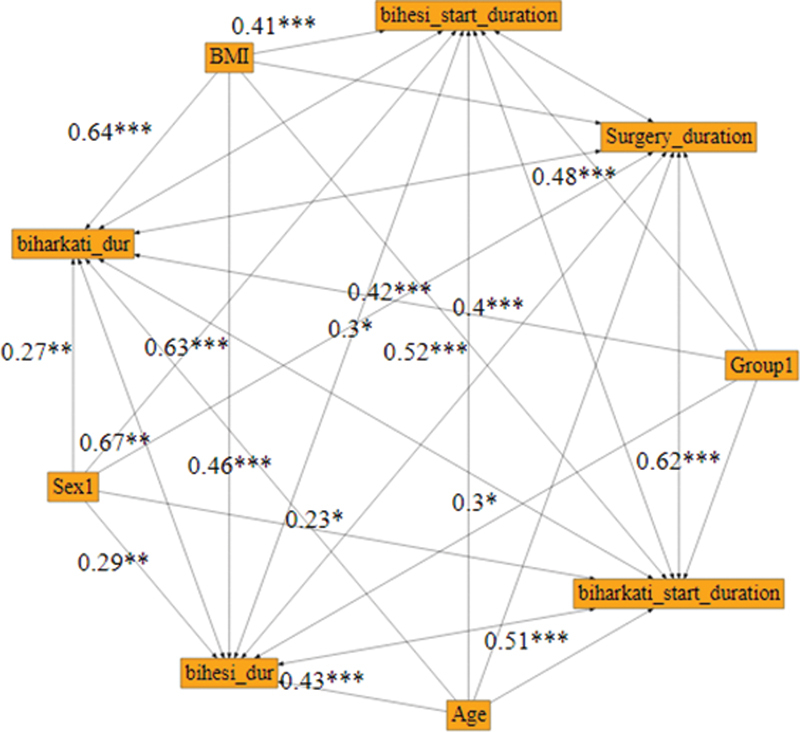
Investigation of the effect of intervention on the dependent variables in the subgroup of biological agents at different times using Bayesian path analysis.

The median time to onset of immobilization in the case group was approximately 4.66 ± 0.909 minutes, which was lengthier than the control group. Furthermore, the variables such as age, gender, and BMI also had a significant effect on the onset time of immobility ( Table 3 and Fig. 1 ).

**Table 3:** Bayesian path analysis output to investigate the effect of intervention variables, age, gender, and BMI on the immobility onset time

Variable	Estimate	SE	Lower CI	Upper CI
Group (ref = control)	4.66	0.909	2.86	6.442
Age	0.118	0.022	0.074	0.161
Sex (ref = male)	1.897	0.886	0.117	3.626
BMI	0.016	0.003	0.01	0.022

Abbreviations: BMI, body mass index; CI, confidence interval; SE, standard error.

### Determination and Comparison of Mean Blood Pressure before Nerve Block in the Two Study Groups

The mean blood pressure before neural block in the case group was estimated to be 3.1 ± 4.941 times higher than the control group. However, the difference did not show any statistical significance. Additionally, there was no significant difference in the mean blood pressure in terms of gender; however, the effects of age and BMI on blood pressure were significant.

### Determination and Comparison of Mean Blood Pressure after Nerve Block in the Two Study Groups

The mean blood pressure after the nerve block for the patients in the case group was estimated to be approximately 0.21 ± 5.031 units lower than the control group, which was not statistically significant . In addition, there was no significant difference in mean blood pressure among gender groups. Nonetheless, the effect of age and BMI on blood pressure was significant.

### Determination and Comparison of Mean Blood Pressure after Surgery in the Two Study Groups

The mean blood pressure after surgery for the case group was estimated to be approximately 1.59 ± 5.14 units lesser than the control group. Statistically, there were no significant differences in this variable. But the effect of age, gender, and BMI on blood pressure was significant.

### Determination and Comparison of Mean Heart Rate before Neural Block in the Two Study Groups

The mean heart rate before the nerve block for the patients in the case group was estimated to be approximately 3.58 ± 4.44 units lesser than the control group. The association was not found to be statistically significant. Also, gender had no significant effect on the heart rate. But the effect of age and BMI on the heart rate was significant.

### Determination and Comparison of Mean Heart Rate after Surgery in the Two Study Groups

The mean heart rate after the surgery for the patients in the case group was approximately 1.02 ± 3.98 units lower than the control group. Considering the obtained validity interval, it can be deduced that the mean heart rate after the surgery did not differ significantly between the case and control groups. Also, gender had no significant effect on the mean heart rate after surgery. But the effect of age and BMI on heart rate was significant.

### Determination and Comparison of Mean Heart Rate 30 minutes after Surgery in the Two Study Groups

The mean heart rate 30 minutes after surgery for the patients in the case group was approximately 0.776 ± 4.58 units higher than the control group. According to the obtained validity interval, it can be deduced that this difference in heart rate between the case and control groups was not statistically significant. But the effect of age, gender, and BMI on heart rate was significant.

### Determination and Comparison of Mean Heart Rate 2 hours after Surgery in the Two Study Groups

**Fig. 2:**
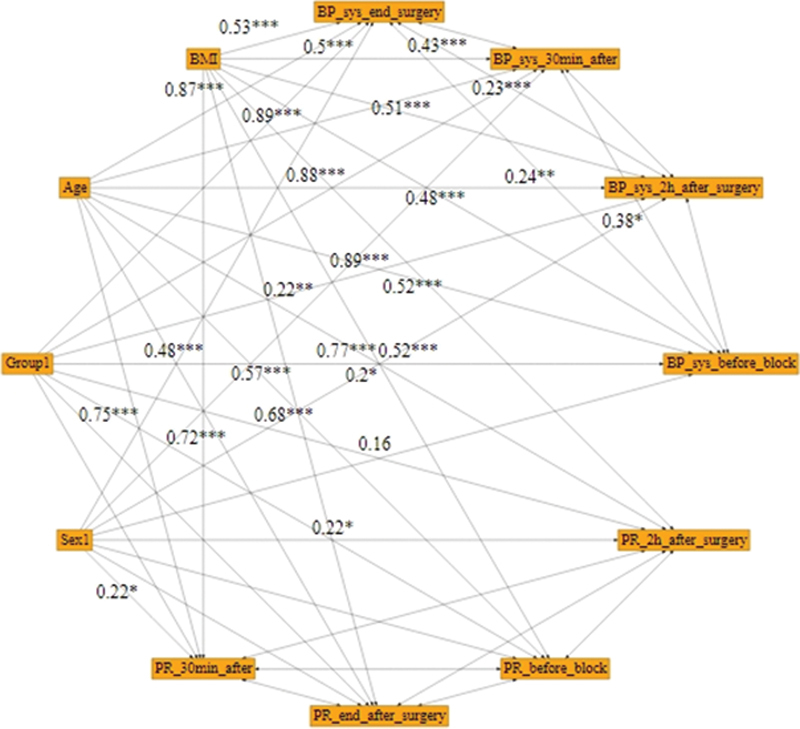
Evaluation of the effect of intervention on pain at different times using Bayesian analysis.

The mean heart rate 2 hours after surgery was approximately 0.39 ± 3.98 more in the case group than in the control group. According to the obtained validity interval, it can be deduced that this difference in heart rate between the two groups is not statistically significant. But the effect of age, gender, and BMI on the heart rate 2 hours after the surgery was significant ( Fig. 2 ).

### Determination and Comparison of Pain 2 hours after Surgery in the Two Study Groups

The mean pain, following 2 hours of the surgery, in the case group was approximately 0.598 ± 0.507 more than in the control group. Based on the obtained credit gap, it can be deduced that this difference is not statistically significant. Also, the age, gender, and BMI had no significant effect.

### Determination and Comparison of Pain 4 Hours after Surgery in the Two Study Groups

The mean pain, following 4 hours of the surgery, was approximately 0.69 ± 0.64 more in the case group than in the control group. Based on the obtained credit gap, it can be deduced that this difference is not statistically significant. Also, gender had no significant effect on pain at 4 hours after surgery. But the effect of age and BMI on pain was significant.

### Determination and Comparison of Pain 6 Hours after Surgery in the Two Study Groups

The mean pain 6 hours after surgery was approximately 0.12 ± 0.765 more in the case group than in the control group. Based on the obtained credit gap, it can be deduced that this difference is not statistically significant. Also, gender had no significant effect on pain at 6 hours after surgery. But the effect of age and BMI on pain was significant at this time.

### Determination and Comparison of Pain Rate 8 Hours after Surgery in the Two Study Groups

The mean pain 8 hours after surgery was approximately 0.64 ± 0.703 units lower in the case group than in the control group. Based on the obtained credit gap, it can be deduced that this difference is not statistically significant. Also, gender had no significant effect on pain at 8 hours after surgery. But the effect of age and BMI on pain was significant at this time.

### Determination and Comparison of Pain Rate 10 Hours after Surgery in the Two Study Groups

The mean pain 10 hours after surgery was approximately 0.61 ± 0.46 units less in the case group than in the control group. Based on the obtained credit gap, it can be deduced that this difference is not statistically significant. Also age had no significant effect on pain at 10 hours after surgery. But the effect of gender and BMI on pain was significant at this time.

### Determination and Comparison of Pain Rate 12 Hours after Surgery in the Two Study Groups

**Fig. 3:**
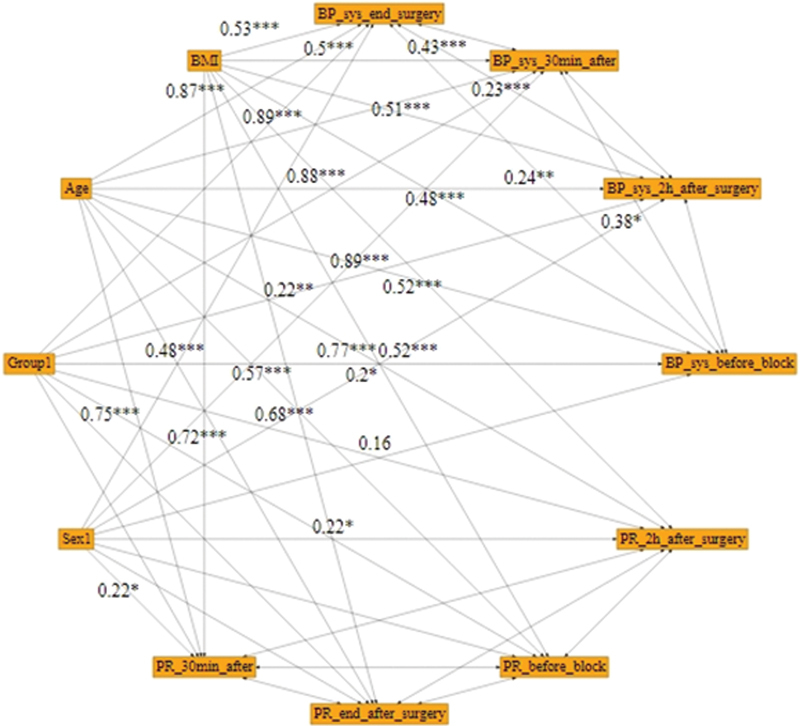
Path analysis for pain-dependent variables at different times.

The mean pain 12 hours after surgery in the case group was approximately 0.88 ± 0.382 units lower than in the control group. Therefore, given the validity gap, it can be deduced that this difference is statistically significant. But age, gender, and BMI had no significant effect on pain at 12 hours after surgery ( Fig. 3 ).

## Discussion

Studies have shown that addition of adjunct magnesium to lidocaine for intravenous regional anesthesia is associated with early onset of sensory block, increased duration of the anesthesia, and low to no side effects. 15 16 In a controlled randomized trial, Haghighi et al 17 found, in 30 patients administered magnesium along with lidocaine for axillary nerve block using the transarterial method, the duration of motor and sensory block to be significantly prolonged 18 19 and invariant with only the lidocaine group. 17 Furthermore, addition of a magnesium adjunct with lidocaine for Bier's block is also reported to decrease chronic limb pain and a number of failed treatments. 20 Our study also reports that at 12 hours following the surgery, the intervention group (magnesium + lidocaine) had reduced incidence of postoperative pain.

Shoeibi et al 21 presented in their study that the use of 10% magnesium sulfate is associated with a significant increase in the duration of spinal anesthesia in patients undergoing cesarean section surgery. 22 In a comparative study, Mirkheshti et al 23 reported that the addition of magnesium to lidocaine for upper extremity surgeries is associated with the increased onset of sensory and motor block and increased block length as compared with the usage of paracetamol with lidocaine.

In a study by Turan et al, 24 in patients undergoing hand surgery under regional anesthesia, addition of 15% magnesium sulfate to lidocaine significantly decreased postoperative pain at 15, 20, 30, 40, and 50 minutes along with a reduced need of diclofenac. Nonetheless, in our study, addition of 50% magnesium sulfate to lidocaine led to a decrease in pain at 2, 6, 8, 10, and 12 hours after surgery. However, this outcome was statistically significant only at 12 hours after surgery. 25 26 Differences in the methods of statistical analysis can be one of the possible causes of the variations in the outcomes.

Our study also revealed that magnesium with lidocaine is not associated with significant unstable changes in hemodynamic parameters such as blood pressure and heart rate. Similar outcomes have been reported by some recent studies conducted for axillary brachial plexus block 27 and laparotomy surgery. 28

## Conclusion

Magnesium sulfate with lidocaine is associated with increased anesthetic and analgesic efficiency with reduced postoperative adverse events. Comparison of various other adjuncts with greater sample sizes can give better inferences.
